# Efficacy of uniportal video assisted thoracoscopic surgery in management of primary spontaneous hemopneumothorax

**DOI:** 10.1016/j.ijscr.2019.01.007

**Published:** 2019-01-19

**Authors:** Yasser M. Aljehani, Jawaher A. Almusairii

**Affiliations:** a Thoracic Surgery Division, Department of Surgery, King Fahad Hospital of the University, Collage of Medicine, Imam Abdulrahman bin Faisal University, Alkhobar 31952, Box 40141, Dammam, Saudi Arabia; b Department of Surgery, King Fahad Hospital of the University, Collage of Medicine, Imam Abdulrahman bin Faisal University, Dammam, Saudi Arabia

**Keywords:** Case report, Primary spontaneous hemopneumothorax, Uniportal VATS

## Abstract

**Introduction:**

Primary spontaneous hemopneumothorax (PSHP) is an accumulation of blood and air in the pleural space without trauma or obvious etiology. It is a rare surgical emergency and may lead to hypovolemic shock if not treated. Early and fast recognition will improve patient outcomes.

**Presentation of the case:**

We present a case of PSHP in a young male utilizing the uniportal video-assisted thoracoscopic surgery (VATS) through the same incisional site of the thoracostomy tube. The patient made an uneventful recovery.

**Discussion:**

VATS has become the first line and the gold standard surgical management of most thoracic surgeries. In case of PSHP, some thoracic surgeons still skeptical about the minimal invasive approach in such emergency. Applying the concept of uniportal technique, which can be used for diagnostic as well as major therapeutic purposes. There is vast literature that support the notion that it reduces postoperative pain and paresthesia and lead to fast patient recovery.

**Conclusion:**

As demonstrated in our case, uniportal VATS is safe and effective in the management of PSHP.

## Highlights

• Primary spontaneous hemopneumothorax is an emergency and should be treated immediatley to prevent any dramatic complication.
• Video assisted thoracoscopic surgery now is the gold standard in treatment of hemopneumothorax.
• Applying the new concept of uniportal technique has a better outcome with fast patient recovery.
• Ability to use thoracostomy tube opening for uniporatal video-assisted thoracoscopic surgery.
• Utilization of uniportal technique even in semi-stable patient is safe and well tolerated.

## 1 Introduction

Primary spontaneous hemopneumothorax (PSHP) is a rare life threatening entity. It is a condition in which blood and air accumulate in the pleural space without obvious etiology or history of trauma [1,2]. It is usually seen in young age group (mean age 27) [3]. Early recognition, is based on the clinical as well as radiological criteria which are the key to management plan [1]. It is one of surgery indications which can be performed either by a thoracotomy or less invasively by Video-Assisted Thoracoscopic Surgery (VATS) [4]. VATS is now becoming less invasive by performing it through only a single incision (Uniportal VATS) [5] This work has been reported in line with the SCARE criteria [6].

## 2 Case report

**Fig. 1:**
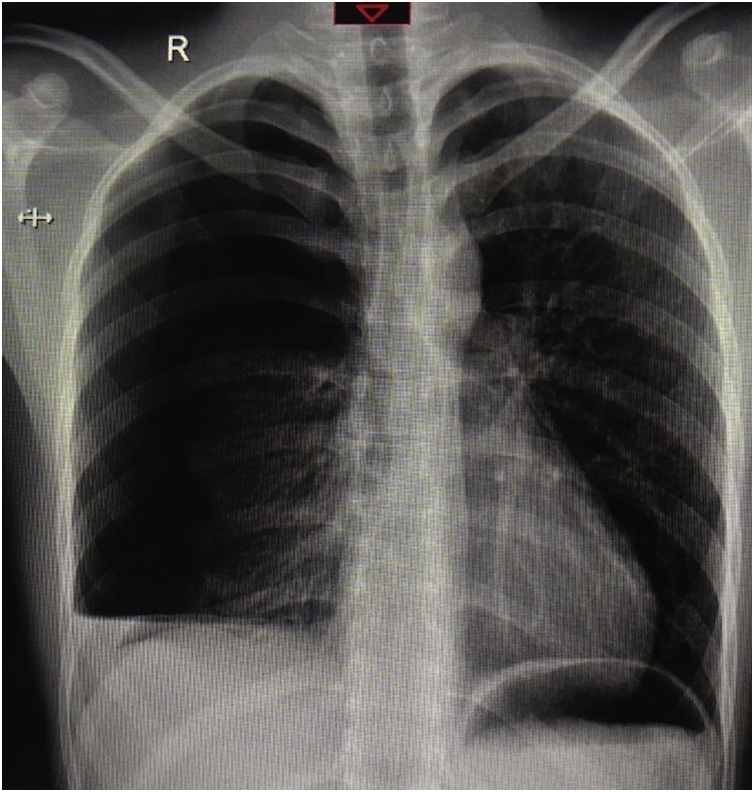
Initial chest x-ray posterior anterior view showed right apical pneumothorax with air fluid level and a collapsed lung.

**Fig. 2:**
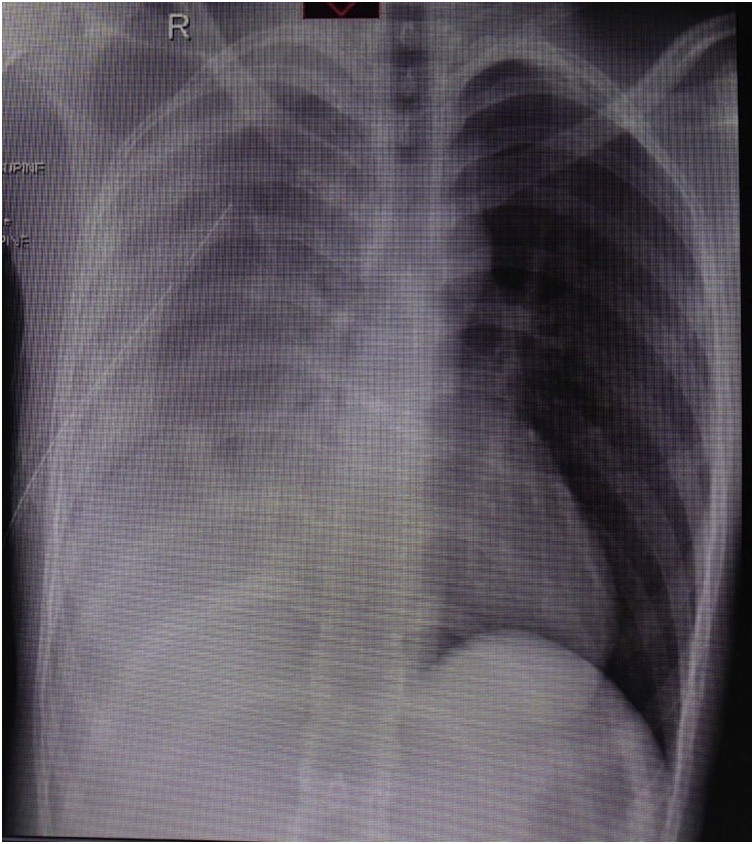
Chest x-ray post thoracostomy tube insertion showed right pneumothorax and opacity.

**Fig. 3:**
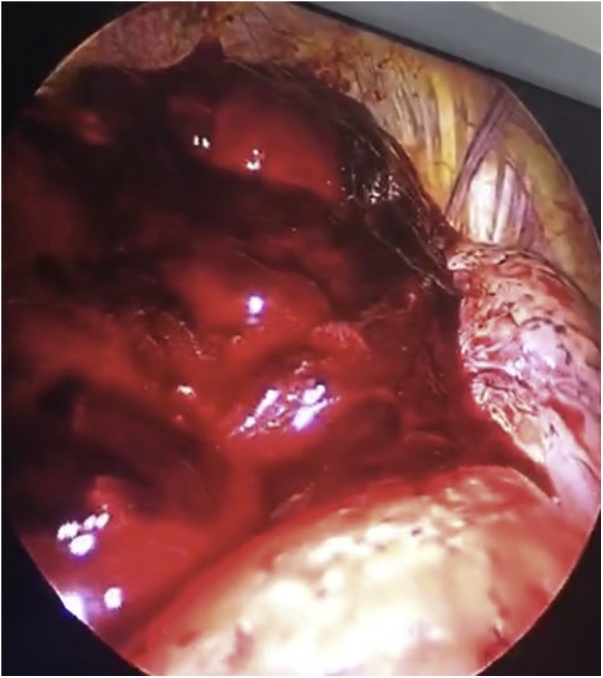
Hematoma that was evacuated from the right pleural cavity.

A 26-year-old male, not known to have any medical illness and not on current medications, who smokes for 8 years 1 pack/day, presented to Emergency Department complaining of a sudden onset of chest pain and shortness of breath for few hours’. There were no other associated symptoms and no history of trauma or any strenuous activity. Drug, family and psychosocial history were negative. No genetic information was available. Upon arrival, he was anxious and ill looking with respiratory distress. His initial vital signs were: Pulse 78 per minute, Blood Pressure(BP) 130/80 mmHg, Temperature 37 °C and oxygen saturation on pulse oximetry 95% (at room air).Chest Auscultation revealed decrees air entry over the right hemithorax and hyper-resonant percussion noted over the same side. Chest radiograph (Fig. 1) showed right apical pneumothorax with air fluid level and a collapsed lung. A decision was taken to insert a thoracostomy tube. A tube (size 32Fr) was inserted in the 5th intercostal space anterior to the mid-axillary line and then it was connected to underwater seal system with suction. Upon insertion of the tube, the initial drainage was more than 500 cc of blood.Routine Laboratory investigation revealed: white blood cells 20.4 × 1000/uL, haemoglobin 11.7 g/dl, hematocrits 34.6٪, Platelets 207 × 1000/uL, PT 12.5 s, PTT INR 1.0.Liver Function Test (LFT) and Renal Function Test (RFT) were within normal limits. Chest radiograph post-thoracostomy tube insertion (Fig. 2) was still showing right pneumothorax and opacity, most likely retained hematoma.Patient condition did not improve over the following hours. He collected almost 1200 cc of blood with persistent tachypnea and respiratory distress over 3 h. A decision was taken by the consultant thoracic surgeon to proceed with operative option, u-VATS. After induction of general anesthesia and double lumen endotracheal tube was inserted, patient was positioned on left lateral decubitus position. A camera 5 mm/30-degree scope was introduced through the already existing thoracostomy tube incision. The pleural cavity explored, a large hematoma was evacuated (Fig. 3). After complete removal of hematoma, exploration was done and there was an active source of bleeding in a vascular adhesion around the subclavian artery which was well controlled by surgical clips. A small bulla was found in the apex of right upper lobe and grasped by endograsper from the same thoracostomy incision, then the apex was resected using stapler device (60 mm covidien^™^) which was also inserted through the same incision. Thoracostomy tube was inserted after that and connected to underwater seal system with continuous suction for 2 days' post-operatively. The procedure was done in accordance to surgical guide and principles which was well tolerated by the patient, he had an uneventful postoperative course without a special consedration and discharged in a stable condition. Chest radiograph at the time of discharge showed complete right lung expansion. The histopathology of the resected specimen showed consistent with bullae with emphysematous changes, inflammation, and hemorrhage. No malignancy.

## 3 Discussion

Primary spontaneous hemopneumothorax (PSHP) is a condition where more than 400 mL of blood accumulates within the pleural space without an obvious etiology, trauma or underlying pulmonary disease [1]. It can be treated conservatively, by thoracostomy tube insertion or surgically by VATS or open thoracotomy [1]. Its major clinical features are chest pain, dyspnea, and sometimes hypovolemic shock, which can be dramatic depends on the amount of hemorrhage [4]. The first reported case of PSHP was in 1879 by Whitaker and it was successfully treated by aspiration [7]. Thoracotomy and decortication of the lung was the first surgical treatment, which was performed by Elrod and Murphy in a patient 6 weeks after the onset of PSHP [8]. VATS has now become the initial and the gold standard treatment for spontaneous hemopneumothorax, especially for patients with active hemorrhage and massive blood clot in the thorax [5]. This kind of procedure leads to less postoperative pain and shorter hospital stay unlike the open thoracotomy which can cause more pain, poor cosmetic outcome, and possible respiratory impairment [5]. It started to evolve from three-ports to double-port and now to uniport technique as how it is performed in our case [9]. The uniportal VATS was first introduced by Gaetano Rocco on 2004 [10]. Since then, it started to be performed for diagnostic and therapeutic purposes through a single incision of 2–2.5 cm long and can be longer or shorter depends on the case [9]. Indications can vary from minor procedures such as pleural diseases to more major procedures such as lobectomies [9]. If the patient presents with a chest drain in place, the same incision can be used to perform the procedure [11]. The main advantage of this minimal invasive procedure is reduction of postoperative pain and paresthesia, therefore, speeding the recovery. This is due to the involvement of only one intercostal space, so one intercostal nerve is likely to be stretched with a single distribution of pain [5].

## 4 Conclusion

The utilization of uniportal VATS in such emergency thoracic entity is safe and well tolerated even in semi-stable patients. Future consideration of this procedure is important since it has better pain control and cosmetic outcome as advantage.

## Conflicts of interest

Authors declare any conflict of interest.

## Funding

No fund is required.

## Ethical approval

Case reports gain exception since our academic institute requires patient’s general consent to all academic and educational material.

## Consent

Written informed consent was obtained from the patient for publication of this case report and accompanying images. A copy of the written consent is available for review by the Editor-in-Chief of this journal on request.

## Author contribution

Aljehani Yasser: Surgeon on charge for the reported case and final reviewer.

Almusairii Jawaher: Data collection and writing the manuscript details.

All authors read and approved the final manuscript.

## Registration of research studies

No registration is required.

## Guarantor

Yasser Aljehani.

## Provenance and peer review

Not commissioned, externally peer-reviewed.
